# The Uncorrected Defect: A Risk Stratification Model for Persistent Levator Ballooning Following Pelvic Organ Prolapse Repair

**DOI:** 10.1007/s00192-026-06662-z

**Published:** 2026-05-04

**Authors:** José Antonio García-Mejido, Sara García-Pombo, Olaya Salas-Álvarez, Ana Fernández-Palacín, Fernando Bugatto-Gonzalez, José Antonio Sainz-Bueno

**Affiliations:** 1 Area of ​​Obstetrics and Gynecology of the Department of Surgery, Faculty of Medicine, University of Seville, Seville, Spain; 2 Biostatistics Unit, Department of Preventive Medicine and Public Health, University of Seville, Seville, Spain; 3 Department of Child and Mother Health and Radiology School of Medicine, University of Cádiz and Division of Maternal-Fetal Medicine, Obstetrics and Gynecology Department, Puerta del Mar University Hospital, Cádiz, Spain; 4 Biomedical Research and Innovation Institute of Cádiz (INiBICA), Cádiz, Spain

**Keywords:** Pelvic organ prolapse, Levator ani muscle, Ballooning, Transperineal ultrasound, Prediction model

## Abstract

**Objective:**

To develop and validate a multivariable prediction model capable of identifying patients at high risk of persistent levator hiatus ballooning 3 months following primary POP reconstructive surgery.

**Method:**

A prospective observational study was conducted on a consecutive cohort of 140 women undergoing primary surgery for symptomatic POP (POP-Q stage ≥ 2). All patients underwent a standardized transperineal ultrasound assessment preoperatively and at 3 months postoperatively. Ballooning was defined as a levator hiatus area (LHA) of ≥ 25 cm^2^ on straining maneuver. A multivariable binary logistic regression model was developed to predict the presence of postoperative ballooning. The model’s performance was assessed using Harrell’s C-index (AUC) and calibration plots.

**Results:**

Although surgery resulted in a significant mean reduction of the LHA on straining (from 29.60 ± 8.30 cm^2^ to 27.17 ± 7.24 cm^2^; *p* < 0.001), the ballooning phenotype persisted in 57.1% (80/140) of the cohort. The final multivariable model identified three independent predictors: age (OR 0.959; 95% CI 0.920–0.999), body mass index (OR 1.095; 95% CI 1.004–1.194), and preoperative resting LHA (OR 1.276; 95% CI 1.158–1.405). Notably, resting area proved to be a stronger predictor than straining area. The model demonstrated high discriminative ability with an AUC of 0.84 (95% CI 0.77–0.91) and good calibration.

**Conclusions:**

Surgical correction of POP reduces hiatal dimensions but fails to correct levator ballooning in the majority of patients. We developed a highly accurate predictive model that identifies this “uncorrected defect” using preoperative resting biometry. Identifying this high-risk phenotype is crucial for patient counseling and managing expectations regarding long-term recurrence.

**Supplementary Information:**

The online version contains supplementary material available at https://doi.org/10.1007/s00192-026-06662-z.

## Introduction

Pelvic organ prolapse (POP) represents a condition with a substantial healthcare burden and a significant impact on women’s quality of life. Corrective surgery is, in fact, one of the most common gynecological procedures, with an estimated lifetime risk ranging between 10% and 20% [1, 2]. Despite the optimization of surgical techniques, the failure of reconstructive surgery remains a primary clinical challenge, evidenced by high reoperation rates.

Current evidence, consolidated through systematic reviews, demonstrates anatomical recurrence rates at levels that could be considered unacceptably high. Although the literature describes broad ranges, from 31% to 59% [3], meta-analyses indicate that the average recurrence rate stabilizes at approximately 36% [4]. Specifically, anatomical recurrence of cystocele (POP-Q stage ≥2) has reached 51.9% in some cohort studies [3]. This nonnegligible scenario of surgical failure underscores the urgent clinical need to optimize our preoperative risk stratification capabilities.

Concurrently, the past decade has witnessed a paradigm shift in pathophysiology. We have transitioned from a classical model focused on fascial defects to an approach that positions the biomechanics of the levator ani muscle (LAM) complex as the key determinant of pelvic support [5–7]. In this context, transperineal ultrasound has emerged as the imaging tool of choice for this functional quantification, demonstrating good reproducibility in diagnosing levator defects [8] and thus allowing the identification of high-risk phenotypes.

Among these phenotypes, levator avulsion and excessive distension of the levator hiatus area (LHA) stand out as the most potent predictors [3, 8, 9]. Avulsion is undoubtedly a robust risk factor, particularly implicated in cystocele recurrence, where it is associated with a relative risk of 2.9 to 4.1 for postoperative anatomical failure [3, 4, 9, 10]. Furthermore, excessive distensibility of the LHA on straining, a phenomenon known as “ballooning” (defined as LHA ≥ 25 cm^2^) [7], has also been independently correlated with the risk of recurrence [1, 3, 4] and with the severity of the prolapse itself [3, 7].

However, the interpretation of preoperative LHA biometry faces a key conceptual dilemma. Current debate centers on whether ballooning is a primary cause of pelvic floor dysfunction or, conversely, a mere mechanical consequence of visceral descent itself, which simply occupies and passively distends the hiatus [7, 11, 12]. This distinction is by no means semantic [11], given that reconstructive surgery itself, when successful, alters LHA biometry.

Indeed, multiple prospective studies using both 3D ultrasound and magnetic resonance imaging consistently confirm that LHA dimensions are significantly reduced following surgical correction [11, 13, 14]. This evidence suggests that a proportion of patients classified with preoperative ballooning may, in reality, have secondary LHA distension—a mechanical consequence of visceral descent that resolves upon repositioning of the structures [5, 11, 12]. The true risk phenotype, therefore, likely resides in those patients who maintain persistent ballooning postoperatively [11], a finding that reflects pathological and intrinsic tissue compliance.

While notable efforts have been made to develop recurrence prediction models based on preoperative variables [3], we still lack tools to discriminate which patients will retain LHA ballooning after intervention. Identifying this high-risk subgroup is, in our view, essential for both patient counseling and advanced surgical planning [3]. We hypothesized that a model integrating baseline clinical characteristics, prolapse typology, and key preoperative ultrasound parameters (such as hiatal biometry and straining dynamics) could reliably identify patients at risk of persistent hiatal biomechanical dysfunction. Consequently, the primary objective of our study was to develop and validate a multivariable mathematical model capable of predicting the presence of LHA ballooning following corrective surgery for POP.

## Material and Method

A prospective observational study was designed and conducted between January 1, 2021, and December 31, 2024, at the University Hospital of Valme (Seville, Spain).

The study protocol was approved by the Biomedical Ethics Committee of Andalucia (Ref 1259-N-20). The research was conducted in adherence to the principles of the Declaration of Helsinki (as revised in 2013), and all participants provided written informed consent prior to inclusion.

### Patient Inclusion

During this period, a consecutive cohort of 140 women scheduled for corrective POP surgery was recruited. The primary inclusion criterion was the presence of symptomatic POP, objectified as stage ≥2 in any compartment according to the POP-Q classification [15], with an indication for primary corrective surgery due to failure or refusal of conservative management. Specifically, all included patients presented with bothersome vaginal bulge symptoms with the leading edge of the prolapse at or beyond the hymen (POP-Q point ≥0). To ensure consistency in surgical technique, all procedures were performed by the same experienced urogynecology surgical team at our institution. Concomitant anatomical defects, such as cervical elongation, were addressed with specific surgical techniques (e.g., the Manchester procedure) at the time of prolapse repair to ensure comprehensive anatomical correction. Exclusion criteria were established as: a history of any prior pelvic reconstructive surgery (whether for prolapse or incontinence) and the presence of known neurological pathology with potential involvement of pelvic floor function.

### Baseline Clinical Assessment

The initial evaluation was carried out in the preoperative consultation by a gynecologist specializing in pelvic floor disorders, following a standardized protocol. Demographic variables (age, menopausal status, body mass index [BMI]) and relevant obstetric history (number of vaginal deliveries and cesarean sections) were collected. Clinical staging of POP was performed systematically with the patient in the dorsal lithotomy position, using the POP-Q system [15]. The maximum stage reached in each compartment (cystocele, uterine prolapse, cervical elongation, rectocele, enterocele) during the execution of a maximal straining maneuver was documented.

### Transperineal Ultrasound Assessment

Ultrasound evaluations were scheduled at two time points: a baseline preoperative assessment (performed after the clinical examination) and a follow-up assessment at 3 months post-surgery. All examinations were performed by a single expert operator, with over a decade of experience in pelvic floor ultrasound. This operator remained blinded to clinical details and the type of surgical intervention at all times. A Toshiba® 700 Aplio ultrasound system (Toshiba Medical Systems Corp., Tokyo, Japan) equipped with an abdominal volumetric probe (PVT-675 MV) was used. The procedure was performed with the patient in dorsal lithotomy, placing the probe on the perineum with minimal pressure. Standardized 3D volumes were obtained under two conditions: at rest and during a sustained maximal straining maneuver (lasting a minimum of 6 s). In accordance with recent terminological recommendations [16], we have used the term “straining” throughout this manuscript (instead of the traditionally used “Valsalva maneuver”) to more accurately describe the voluntary increase in intra-abdominal pressure used to assess levator hiatus distensibility.

Analysis of the acquired 3D volumes was performed offline by the same blinded examiner. The plane of minimal dimensions (PMD) was identified, defined as the axial plane showing the shortest distance between the postero-inferior border of the pubic symphysis and the anterior border of the LAM. In the PMD, the LHA (in cm^2^) was measured both at rest and during maximal straining. Consequently, ballooning was defined as an LHA ≥ 25.0 cm^2^ on maximum straining, as previously defined by Dietz et al. [7].

### Statistical Analysis

Statistical data processing was performed entirely using the IBM SPSS Statistics package, version 31 for Windows (IBM Corp., Armonk, NY, USA). A statistical significance level of *p* <.05 was set for all two-sided tests.

The sample size was determined a priori to detect a clinically relevant difference of 8% in the paired proportions of levator ballooning presence (preoperative vs postoperative). Assuming a two-sided alpha error of 0.05 and a statistical power of 80%, a minimum of 135 patients was required. A total of 140 women were recruited to account for potential data loss.

Initially, the normality of quantitative variables was assessed using the Shapiro–Wilk test. Variables with a normal distribution were presented as mean ± standard deviation; with marked asymmetry variables expressed as median and interquartile range. Absolute frequencies and percentages were used for qualitative or categorical variables, and 95% confidence intervals (CI) were calculated for the main descriptive parameters.

In the bivariate analysis, the comparison of means between independent groups was performed using the Student’s *t*-test for independent samples. In the case of non-normal variables, the Mann–Whitney *U* test was applied. For the comparison of proportions between categorical variables, the chi-square (χ^2^) test was used, resorting to Fisher’s exact test for 2 × 2 tables, when the percentage of expected frequencies were < 5 exceeded 25%.

To assess the impact of surgery, ultrasound measurements (such as levator hiatus area at rest and straining) collected consistently at 3 months post-surgery were compared before and after the intervention. For this purpose, the paired Student’s *t*-test or the Wilcoxon signed-rank test was used, depending on whether the data were normally distributed.

Finally, a multivariable binary logistic regression model was constructed to identify independent predictors of postoperative ballooning. The discriminative ability of said model was determined by calculating Harrell’s C-index (equivalent to the area under the receiver operating characteristic curve, AUC of the probabilities estimated by the model), with its 95% CI. Model calibration, understood as the agreement between predicted and observed probabilities, was assessed visually via a calibration plot and the calculation of the calibration slope.

## Results

The final study cohort comprised 140 patients scheduled for surgical correction of pelvic organ prolapse. Baseline demographic and clinical characteristics are detailed in Table 1. The specific types of reconstructive surgeries performed, including the anatomical compartments repaired and concomitant procedures, are detailed in Table 1. The mean age was 56.7 ± 10.12 years; 60.7% (85/140) were postmenopausal. The mean BMI was 27.53 ± 4.93 kg/m^2^, with a mean parity of 2.29 ± 1.02 deliveries.

**Table 1:** Baseline demographic and clinical characteristics of the study population

General characteristics (*n* = 140)	Value	95% CI
Age (years)	56.70±10.12	55.01; 58.39
Menopause	85/140 (60.7%)	52.4%; 68.4%
Age at menopause (years)	50.32±4.08	49.45; 51.18
Weight (kg)	71.66±12.76	69.53; 73.79
Height (m)	1.61±0.07	1.60; 1.63
BMI **(kg/m**^**2**^**)**	27.53±4.93	26.71; 28.36
Obstetric history		
Parity	2.29±1.02	2.12; 2.46
Cesarean sections	0.12±0.42	0.05; 0.19
Presence of cystocele	92/140 (65.7%)	57.5%; 73.1%
Stage I	7/140 (5%)	
Stage II	19/140 (13.6%)	
Stage III	66/140 (47.1%)	
Presence of uterine prolapse	36/140 (25.7%)	19.2%; 33.6%
Stage I	5/140 (3.6%)	
Stage II	9/140 (6.4%)	
Stage III	20/140 (14.3%)	
Stage IV	2/140 (1.4%)	
Presence of cervical elongation	84/140 (60%)	51.7%; 67.7%
Stage I	2/140 (1.4%)	
Stage II	18/140 (12.9%)	
Stage III	64/140 (45.7%)	
Presence of rectocele	40/140 (28.6%)	21.7%; 36.6%
Stage I	15/140 (10.7%)	
Stage II	17/140 (12.1%)	
Stage III	8/140 (28.6%)	
Presence of enterocele	6/140 (4.3%)	1.8%; 9.2%
Stage I	0/140 (0%)	
Stage II	4/140 (2.9%)	
Stage III	2/140 (1.4%)	
Surgical procedures		
Anterior colporrhaphy	82/140 (58.6 %)	
Apical suspension (Richter sacrospinous fixation)	4/140 (2.9 %)	
Manchester procedure	81/140 (57.9 %)	
Vaginal hysterectomy	34/140 (24.3 %)	
Posterior compartment repair (including perineorrhaphy)	17/140 (12.1 %)	
Levator plication	0/140 (0 %)	

Values are presented as mean ± standard deviation or *n* (%). *BMI* Body mass index; *CI* Confidence interval

When analyzing the overall effect of surgery in the complete cohort (*n* = 140), we observed a statistically significant reduction in the prevalence of ballooning, decreasing from 69.3% (preoperative) to 57.1% (postoperative) (*p* = 0.012; 95% CI 3.1–20.8). This change was reflected in LHA dimensions (Table 2), where a significant mean reduction in hiatal area during straining was confirmed postoperatively, decreasing from 29.60 ± 8.30 cm^2^ to 27.17 ± 7.24 cm^2^ (*p* < 0.001).

**Table 2:** Ultrasound measurements before and after surgery

	Ultrasound evaluation before surgery (*n* = 140)	Ultrasound evaluation after surgery (*n* = 140)	*p* value	95% CI
Balloning	97/140 (69.3%)	80/140 (57.1%)	0.012	3.1%; 20.8%
**Resting LHA (cm**^**2**^**)**	23.42±6.08	22.83±6.14	0.223	−0.36; 1.54
Straining **LHA (cm**^**2**^**)**	29.60±8.30	27.17±7.24	<0.001	1.31; 3.54

Data are presented as *n* (%) or mean ± standard deviation

Table 3 compares patient characteristics according to the presence of ballooning. Women with higher parity were more likely to present with preoperative ballooning (2.39 ± 0.91 vs 2.05 ± 1.21; *p* = 0.016) and a lower mean number of cesarean sections (0.06 ± 0.28 vs 0.26 ± 0.62; *p* = 0.011) compared to those without it (*n* = 43). Similarly, younger age was significantly associated with the persistence of postoperative ballooning (mean age 54.79 ± 9.96 vs 59.25 ± 9.84 years; *p* = 0.011) and had a higher body weight (73.81 ± 12.93 vs 68.80 ± 12.03 kg; *p* = 0.017) than those without postoperative ballooning.

**Table 3:** Patient characteristics stratified by the presence of ballooning before and after surgery

	With ballooning before surgery (*n* = 97)	Without ballooning before surgery (*n* = 43)	*p* value (P1)	95% CI (1)	With ballooning after surgery (*n* = 80)	Without ballooning after surgery (*n* = 60)	*p*-value (P2)	95% CI (2)
Age (years)	55.93±9.60	58.44±11.12	0.176	−6.17;1.14	54.79±9.96	59.25±9.84	0.011	−8.00;−1.00
Age at menopause (years)	50.66±3.67	49.62±4.81	0.263	−0.79;2.87	50.12±4.18	50.51±4.02	0.591	−2.00;1.00
Weight (kg)	72.01±12.57	70.88±13.29	0.509	−3.00;6.00	73.81±12.93	68.80±12.03	0.017	1.00;9.00
Height (m)	1.62±0.6	1.61±0.08	0.356	−0.01;0.04	1.62±0.06	1.60±0.07	0.109	−0.004;0.04
BMI	27.55±4.95	27.49±4.95	0.962	−1.65;1.70	28.12±5.28	26.74±4.34	0.202	−0.58;2.72
Obstetric history								
Parity	2.39±0.91	2.05±1.21	0.016	0.001;1.00	2.29±0.86	2.28±1.21	0.690	−0.001;0.001
Cesarean sections	0.06±0.28	0.26±0.62	0.011	0.001;0.002	0.10±0.34	0.15±0.52	0.773	−0.001;0.002

Data are expressed as mean ± standard deviation. **P1**: Comparison between patients *with* preoperative ballooning vs *without* preoperative ballooning. **P2**: Comparison between patients *with* postoperative ballooning vs *without* postoperative ballooning. **95% CI**: 95% confidence interval of the difference

Ultrasound measurements (detailed in Table 4) confirmed that the preoperative ballooning group (*n* = 97) presented significantly larger baseline hiatal areas than the group without ballooning (*n* = 43), both at rest (25.72 ± 5.67 cm^2^ vs 18.24 ± 3.06 cm^2^; *p* < 0.001) and on straining (33.64 ± 6.49 cm^2^ vs 20.47 ± 2.94 cm^2^; *p* < 0.001). Notably, this difference between baseline groups persisted after intervention; the preoperative ballooning group continued to record larger postoperative areas at rest (24.22 ± 5.93 cm^2^ vs 19.69 ± 5.47 cm^2^; *p* < 0.001) and on straining (29.30 ± 6.57 cm^2^ vs 22.36 ± 6.37 cm^2^; *p* < 0.001).

**Table 4:** Measured levator hiatus area according to the presence or absence of ballooning before and after surgery

	With ballooning before surgery (*n* = 97)	Without ballooning before surgery (*n* = 43)	*p* value (P1)	95% CI (1)	With ballooning after surgery (*n* = 80)	Without ballooning after surgery (*n* = 60)	*p* value (P2)	95% CI (2)
Preoperative resting LHA	25.72±5.67	18.24±3.06	<0.001	5.02;8.50	26.88±5.72	19.97±4.84	<0.001	4.28;7.52
Preoperative straining LHA	33.64±6.49	20.47±2.94	<0.001	10.50;14.00	32.95±8.01	25.11±6.36	<0.001	5.00;10.00
Postoperative resting LHA	24.22±5.93	19.69±5.47	<0.001	2.03;6.46	26.47±5.04	17.98±3.60	<0.001	6.86;9.54
Postoperative straining LHA	29.30±6.57	22.36±6.37	<0.001	4.48;9.07	32.06±5.09	20.64±3.59	<0.001	9.08;12.00

Data are expressed as mean ± standard deviation (cm^2^). **P1**: Comparison between patients *with* preoperative ballooning vs *without* preoperative ballooning. **P2**: Comparison between patients *with* postoperative ballooning vs *without* postoperative ballooning

Regarding predictors of the outcome, patients who ended up with postoperative ballooning (*n* = 80) started with significantly larger preoperative hiatal areas compared to those who did not (*n* = 60). Specifically, preoperative resting area was 26.88 ± 5.72 cm^2^ vs 19.97 ± 4.84 cm^2^ (*p* < 0.001), and straining area was 32.95 ± 8.01 cm^2^ vs 25.11 ± 6.36 cm^2^ (*p* < 0.001). As a postoperative outcome, this group (*n* = 80) maintained a resting area of 26.47 ± 5.04 cm^2^ (vs 17.98 ± 3.60 cm^2^; *p* < 0.001) and a straining area of 32.06 ± 5.09 cm^2^ (vs 20.64 ± 3.59 cm^2^; *p* < 0.001).

Finally, Table 5 quantifies the net effect of surgery (the pre–post difference) on LHA. In patients with preoperative ballooning (*n* = 97), surgery achieved a mean reduction in straining LHA of 4.33 ± 6.09 cm^2^. In contrast, in patients without baseline ballooning (*n* = 43), LHA area increased slightly by a mean of 1.88 ± 5.90 cm^2^ (*p* < 0.001). This same pattern was observed at rest (reduction of 1.49 cm^2^ vs increase of 1.45 cm^2^; *p* = 0.005). Viewed from the outcome perspective, patients without postoperative ballooning (*n* = 60) experienced a significantly greater mean reduction in straining LHA compared to those who did present it (*n* = 80) (4.47 ± 5.54 cm^2^ vs 0.89 ± 7.05 cm^2^; *p* = 0.001). The pattern was repeated in resting measurements (*p* = 0.007).

**Table 5:** Differences in the measurements of the levator hiatus area before and after surgery according to the presence or absence of ballooning

	With ballooning before surgery (*n* = 97)	Without ballooning before surgery (*n* = 43)	*p* value (P1)	95% CI (1)	With ballooning after surgery (*n* = 80)	Without ballooning after surgery (*n* = 60)	*p* value (P2)	95% CI (2)
Change in resting LHA (pre- vs post-surgery)	1.49±5.94	−1.45±4.51	0.005	0.90;4.56	−0.46±5.96	1.99±5.02	0.007	−3.80;−0.56
Change in straining LHA (pre- vs post-surgery)	4.33±6.09	−1.88±5.90	<0.001	4.03;8.41	0.89±7.05	4.47±5.54	0.001	−5.75;−1.40

Data are expressed as mean ± standard deviation. **P1**: Comparison between patients *with* preoperative ballooning vs *without* preoperative ballooning. **P2**: Comparison between patients *with* postoperative ballooning vs *without* postoperative ballooning

### Postoperative Ballooning Prediction Model

A binary logistic regression strategy was employed to develop the prediction model for postoperative ballooning. The process began with a bivariate analysis to identify candidate variables (selecting those with *p* < 0.20), which included age, BMI, and preoperative resting LHA. Subsequently, these variables were entered into a multivariable analysis, selecting the final model based on a balance between maximum discriminative ability, good calibration, parsimony, and clinical interpretability.

The components of the final multivariable model are detailed in Table 6. The variables that maintained a statistically significant and independent association with postoperative ballooning were age (odds ratio [OR] 0.959; 95% CI 0.920–0.999; *p* = 0.043), BMI (OR 1.095; 95% CI 1.004–1.194; *p* = 0.039), and preoperative resting area (OR 1.276; 95% CI 1.158–1.405; *p* < 0.001).

**Table 6:** Final multivariate logistic regression model for the prediction of postsurgical ballooning

Variables in the model	Odds ratio (OR)	95% CI	*p* value
Age (years)	0.959	0.920; 0.999	0.043
BMI (kg/m^2^)	1.095	1.004; 1.194	0.039
Preoperative resting LHA (cm^2^)	1.276	1.158; 1.405	<0.001

*LHA* Levator hiatus area; *BMI* Body mass index; *CI* Confidence interval

On the basis of the coefficients of this model, the following formula was generated to estimate the probability of postoperative ballooning:

$$\mathrm{Probability}\;\mathrm{of}\;\mathrm{postoperative}\;\mathrm{ballooning}=1/\left(1+\mathrm e\,\,\widehat{\phantom{-}}-\left(-5.309-\left(0.042\;\ast\;\mathrm{age}\right)+\left(0.091\;\ast\;\mathrm{BMI}\right)+\left(0.244\ast\;\mathrm{preoperative}\;\mathrm{resting}\;\mathrm{LHA}\right)\right)\right)$$

To develop the predictive model, bivariate logistic regression was initially performed. Variables demonstrating an association with a *p* value < 0.20 (age, BMI, and preoperative resting LHA) were subsequently entered into a multivariable binary logistic regression model. The final model was optimized for maximum discriminatory capability (assessed via Harrell’s C-index and ROC curve AUC), calibration, and model parsimony. The predictive equation was derived using the standard logistic regression formula based on the final model’s coefficients.

**Fig. 1:**
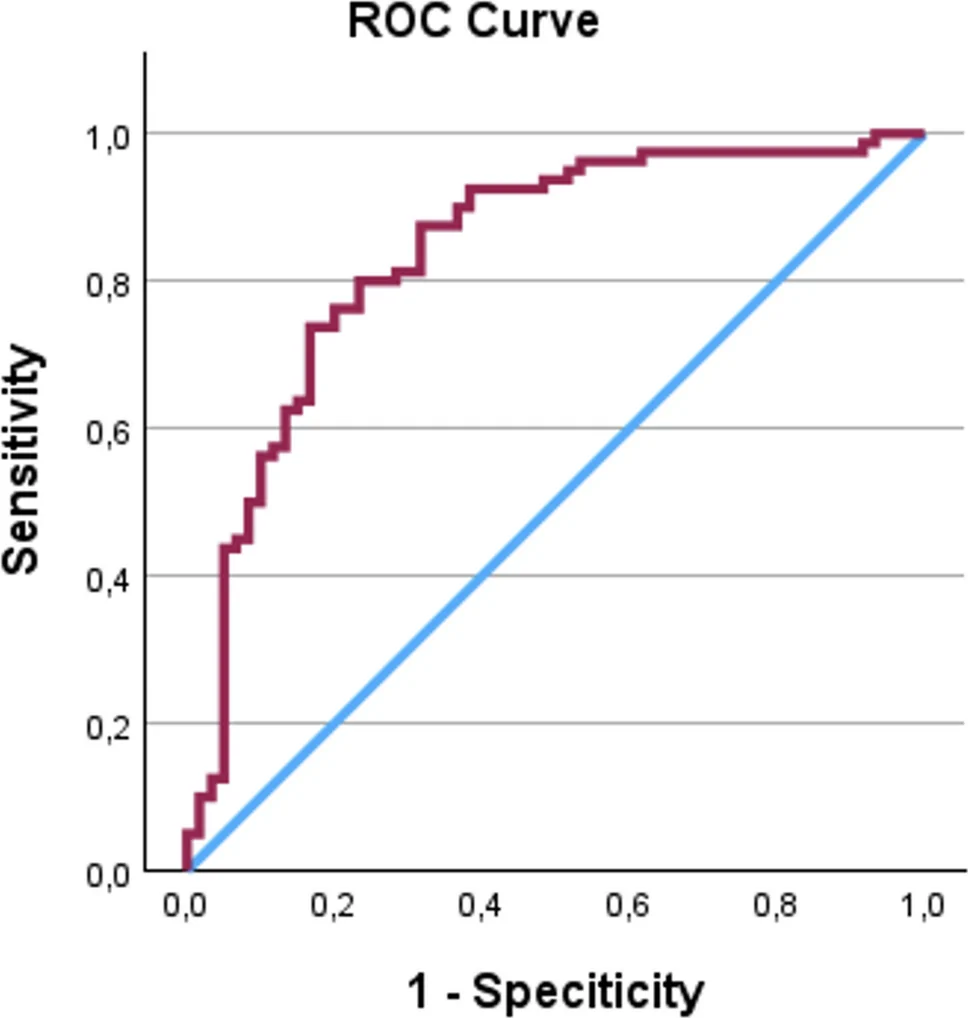
Receiver operating characteristic (ROC) curve of the final model for predicting postsurgical ballooning. The area under the curve (Harrell’s C-index) was 0.84 (95% CI 0.77–0.91; *p* < 0.0005)

**Fig. 2:**
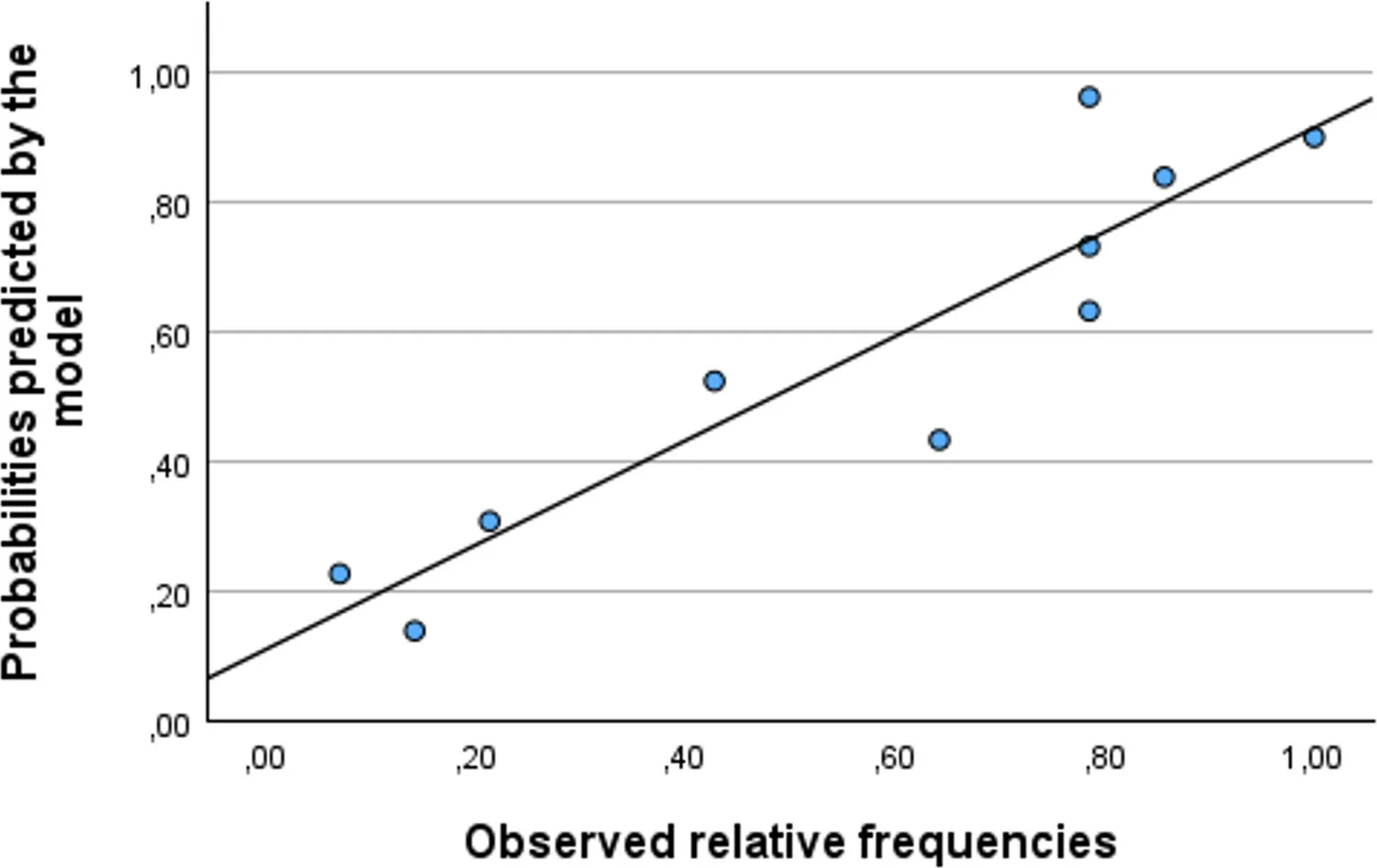
Calibration plot of the predictive model. The plot compares the predicted probability of postsurgical ballooning (X-axis) with the observed frequency (Y-axis). The calibration slope was 0.81 (95% CI 0.53–1.0)

Assessment of the model’s diagnostic performance yielded good discriminative ability, with a Harrell’s C-index of 0.84 (95% CI 0.77–0.91; *p* < 0.0005). The corresponding receiver operating characteristic (ROC) curve is presented in Fig. 1. Model calibration, assessed visually via the calibration plot (Fig. 2), was considered adequate, showing a slope of 0.81 (95% CI 0.53–1.09; *p* < 0.001).

**Fig. 3:**
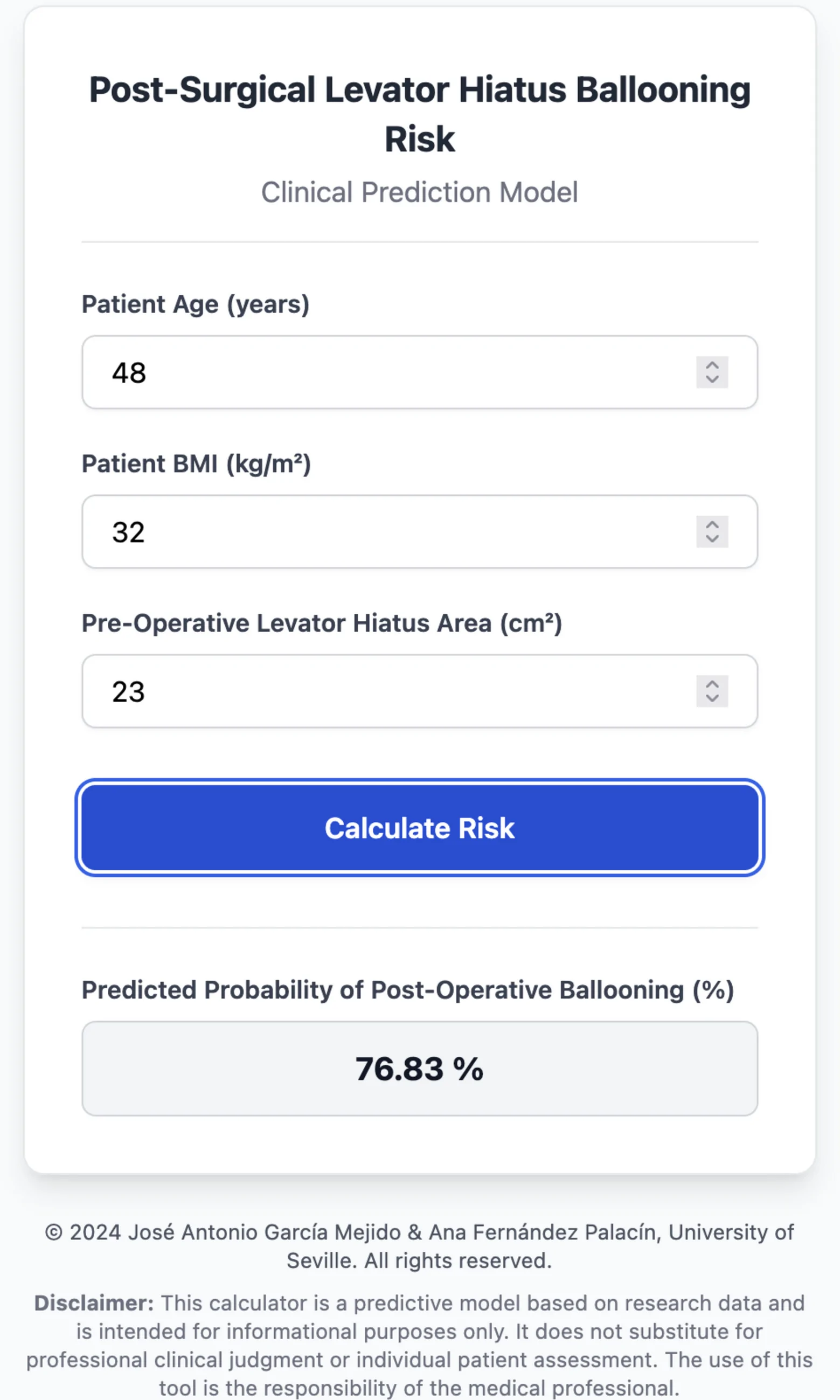
Demonstration of the software application. Data entry for a hypothetical patient (48 years old, BMI 32 kg/m^2^, and preoperative resting LHA 23 cm^2^) yields a predicted probability of postoperative ballooning of 76.83%

Finally, Fig. 3 and Supplementary Video 1 offer a demonstration of the application of this model and the developed software.

## Discussion

This prospective study shows that corrective pelvic floor surgery induces significant and quantifiable anatomical modifications in the levator hiatus area (LHA). Our most immediate finding is the confirmation of a statistically significant reduction in the LHA, assessed via transperineal ultrasound, both under resting conditions and during the straining maneuver. While we observed a decrease in the mean resting LHA from 23.42±6.08 cm^2^ to 22.83±6.14 cm^2^, this change did not reach statistical significance (*p* = 0.223). However, a marked and significant reduction was observed in straining LHA, decreasing from 29.60±8.30 cm^2^ to 27.17±7.24 cm^2^ following the intervention (*p* < 0.001). Parallel to this biometric remodeling, we noted a significant decrease in the prevalence of ballooning, which dropped from 69.3% (97/140) in the preoperative cohort to 57.1% (80/140) at the postoperative control (*p* = 0.038). However, beyond these population-level anatomical changes, the core contribution of our work lies in the observation that ballooning persists in a majority of patients (57.1%) and, fundamentally, in the generation of a prediction model capable of discriminating which women will present this persistence. We consider this development to be the most clinically relevant result of the study.

Our preoperative findings, which link a larger LHA with the presence of ballooning, support the conceptualization of this phenomenon as an ultrasound marker of excessive distension and, ultimately, as the clinical manifestation of underlying hiatal pathology [7, 17, 18]. Likewise, the reduction in LHA dimensions following POP correction is consistent with previous evidence regarding the morphological impact of surgery [11, 13, 14]. However, our data uncover a profound clinical challenge: the high rate of ballooning persistence (57.1%) despite intervention. This fact is crucial, given that the literature consistently identifies ballooning [1, 4, 19] and concomitant avulsion [4, 9, 10, 20] as the dominant risk factors for recurrence. Our data suggest, therefore, that while surgery succeeds in reducing global dimensions [11, 13, 21], it fails to correct the fundamental hiatal pathology in a large proportion of patients.

In this regard, the model we propose to estimate the probability of postoperative ballooning differs qualitatively from those designed to predict global anatomical recurrence [3, 22]. Its value lies in focusing specifically on the persistent ballooning phenotype, understanding it as a surrogate marker for high risk of surgical failure [3, 4]. Although surgery reduces the absolute hiatal area [11, 13, 14], the persistence of ballooning in 57.1% of the cohort [11] implies that these patients maintain significantly dilated postoperative hiatuses, a biometry that is intrinsically linked to a higher risk of POP recurrence [1, 4, 19]. Remarkably, resting area proved to be a better predictor than straining area. This may be because the resting measurement reflects basal structural muscle damage without the mechanical bias of visceral descent, which passively distends the hiatus during effort and could mask true muscle distensibility.

### Strengths and Limitations

The main strength of this work is the development of a novel prognostic model. Unlike previous models focused on general anatomical recurrence [3, 22], ours focuses on a specific ultrasound phenotype (persistent ballooning) as a method to stratify a population with a particular preoperative risk.

However, the study presents key limitations. The most important is that the model requires rigorous external validation, as it was developed and internally validated in the same cohort. Likewise, the current model is based solely on passive biometry (rest and straining) and does not incorporate critical confounding variables, identified by the literature as determinants, especially levator avulsion [4, 9, 10]. Another limitation is that, given the high prevalence of multi-compartment prolapse, most patients underwent combined surgical procedures. Consequently, the study was not powered to isolate and compare which specific surgical technique was superior in reducing the LHA.

The most relevant clinical implication of our study lies not in the mere observation that the LHA is reduced, but in the demonstration that ballooning persists in the majority of cases and, crucially, that this postoperative state is predictable. From a pragmatic perspective, our model offers a prognostic tool that complements other established models [3, 19], but with a specific focus on predicting persistent ballooning.

The ability to preoperatively identify patients at elevated risk of persistent ballooning allows for much more precise counseling. Furthermore, it could play a role in the selection of the surgical technique [3, 4], as postoperative ballooning must be considered a clinically relevant finding. This group of patients (57% in our cohort) may require closer follow-up, as they represent a high-risk phenotype for recurrence [4, 7, 19]. This information is fundamental for counseling, especially in women who present with markedly distended hiatuses at baseline [1, 3, 4, 23].

Regarding future research, external validation of our prediction model is imperative. It is fundamental to distinguish between ballooning persistence (an ultrasound finding) and clinical failure. Our model effectively identifies the risk of persistent biomechanical alteration; the next imperative step is to confirm, through long-term follow-up, the extent to which this risk phenotype translates into actual symptomatic recurrence. Such studies must necessarily include the assessment of levator avulsion status [3, 4, 9, 10] and muscle contractile function [10, 17] as essential covariates to refine predictive power. Additionally, while our current model focused on demographic and sonographic predictors, future studies should explore whether specific preoperative POP-Q stages or affected compartments independently influence the persistence of postoperative levator ballooning. Our data, in line with other authors, suggest that simple passive reduction of the hiatus is not synonymous with functional restoration [11, 21, 23].

## Conclusions

In conclusion, this prospective study demonstrates that while corrective pelvic floor surgery significantly reduces LHA dimensions, ballooning persists in the majority of operated patients. The main finding is the development of a model capable of predicting the probability of this postoperative ballooning, allowing for the identification of a high-risk subgroup that is not discernible solely through preoperative biometry.

## Supplementary Information

Below is the link to the electronic supplementary material.

Supplementary Video 1: Demonstration of the software application. (MOV 6644 KB)
